# Isolation of Genetically Diverse Marburg Viruses from Egyptian Fruit Bats

**DOI:** 10.1371/journal.ppat.1000536

**Published:** 2009-07-31

**Authors:** Jonathan S. Towner, Brian R. Amman, Tara K. Sealy, Serena A. Reeder Carroll, James A. Comer, Alan Kemp, Robert Swanepoel, Christopher D. Paddock, Stephen Balinandi, Marina L. Khristova, Pierre B. H. Formenty, Cesar G. Albarino, David M. Miller, Zachary D. Reed, John T. Kayiwa, James N. Mills, Deborah L. Cannon, Patricia W. Greer, Emmanuel Byaruhanga, Eileen C. Farnon, Patrick Atimnedi, Samuel Okware, Edward Katongole-Mbidde, Robert Downing, Jordan W. Tappero, Sherif R. Zaki, Thomas G. Ksiazek, Stuart T. Nichol, Pierre E. Rollin

**Affiliations:** 1 Special Pathogens Branch, Centers for Disease Control and Prevention, Atlanta, Georgia, United States of America; 2 National Institute for Communicable Diseases, Special Pathogens Unit, Johannesburg, South Africa; 3 Infectious Disease Pathology Branch, Centers for Disease Control and Prevention, Atlanta, Georgia, United States of America; 4 Global AIDS Program, Centers for Disease Control and Prevention, Entebbe, Uganda; 5 Biotechnology Core Facility Branch, Centers for Disease Control and Prevention, Atlanta, Georgia, United States of America; 6 Epidemic and Pandemic Alert and Response Department, World Health Organization, Geneva, Switzerland; 7 Uganda Virus Research Institute, Entebbe, Uganda; 8 Ibanda District Hospital, Ibanda, Uganda; 9 Uganda Wildlife Authority, Kampala, Uganda; 10 Ministry of Health, Republic of Uganda, Kampala, Uganda; Erasmus Medical Center, The Netherlands

## Abstract

In July and September 2007, miners working in Kitaka Cave, Uganda, were diagnosed with Marburg hemorrhagic fever. The likely source of infection in the cave was Egyptian fruit bats (*Rousettus aegyptiacus*) based on detection of Marburg virus RNA in 31/611 (5.1%) bats, virus-specific antibody in bat sera, and isolation of genetically diverse virus from bat tissues. The virus isolates were collected nine months apart, demonstrating long-term virus circulation. The bat colony was estimated to be over 100,000 animals using mark and re-capture methods, predicting the presence of over 5,000 virus-infected bats. The genetically diverse virus genome sequences from bats and miners closely matched. These data indicate common Egyptian fruit bats can represent a major natural reservoir and source of Marburg virus with potential for spillover into humans.

## Author Summary

Marburg virus, similar to its close cousin Ebola virus, can cause large outbreaks of hemorrhagic fever (HF) in rural Africa with case fatalities approaching 90%. For decades, a long-standing enigma has been the identity of the natural reservoir of this deadly virus. In this report, we identify the cave-dwelling Egyptian fruit bat (*Rousettus aegyptiacus*) as a natural host of Marburg virus based on multiple lines of evidence which include, for the first time ever, the isolation of virus directly from wild-caught and apparently healthy bats. The species *R. aegyptiacus* is common throughout Africa with distribution into the eastern Mediterranean and Middle East. Our finding of active virus infection in approximately 5% of *R. aegyptiacus* bats and their population exceeding 100,000 in Kitaka cave in Uganda suggests there are likely over 5,000 Marburg virus–infected bats in this cave, which is only one of many such cave populations throughout Africa. Clearly, these bats could serve as a major source of virus with potential to initiate human epidemics, and the implications for public health are striking. Additionally, we found highly divergent (21%) genome sequences among viruses circulating in these bat populations, a level of diversity that would result from a long-term association with a suitable reservoir host of large population size.

## Introduction

Viruses of the *Marburgvirus* and *Ebolavirus* genera (family *Filoviridae*) cause outbreaks of hemorrhagic fever in Africa characterized by person-to-person spread and high case fatality. Humans have on occasion acquired infection from contact with tissues of diseased nonhuman primates and perhaps herbivores, but the susceptibility of these animals to fatal infection renders it unlikely that they could serve as filoviruses reservoir hosts.

Although the source of filoviruses in nature has not been definitively identified, the cumulative evidence suggests that bats are involved. The infected monkeys consigned from Uganda to Europe in 1967, which resulted in the first recognized outbreaks of Marburg hemorrhagic fever (MHF), were caught on the shores of Lake Victoria and on islands where fruit bats are prevalent [1]. In 1975, the second recorded outbreak of MHF involved tourists who slept at two locations in Zimbabwe in rooms containing insectivorous bats followed by a purported visit to Chinhoyi caves (formerly Sinoia caves) where bats may also have been present [2]. In the first recognized outbreak of Ebola hemorrhagic fever (EHF) in 1976, the first six patients worked in a room where bats roosted in a cotton factory in Sudan [3]. In 1980 and 1987, two patients who developed MHF in Kenya both visited a cave inhabited by bats shortly before becoming ill [4],[5]. In 1994, chimpanzees which developed EHF in Cote d'Ivoire had been observed feeding in a wild fig tree together with fruit bats for two weeks before developing the disease [6]. The Reston ebolavirus, which is apparently nonpathogenic for humans, was introduced into the USA and Europe on several occasions via imported infected monkeys from the Philippines, and each time the animals originated from a single export facility located on the grounds of a former fruit orchard where they were potentially exposed to the excreta of fruit bats [7]. In1996, it was shown that experimentally infected fruit bats were capable of supporting replication of ebolavirus without developing overt disease [8]. In 1998–2000, a protracted outbreak of MHF in Durba village in northeastern Democratic Republic of the Congo (DRC) consisted of repeated occurrences of short transmission chains arising in workers in Goroumbwa Mine where large numbers of bats roosted. The impression that there were recurrent introductions of infection into humans from a natural source was supported by finding that multiple genetic lineages of virus circulated during the outbreak [9]. Significantly, diverse genetic lineages of Marburg virus were detected in Egyptian fruit bats, *Rousettus aegyptiacus*, and two species of insectivorous bat in the mine, and the outbreak ceased when the mine flooded, but no live virus was isolated from bats [10]. In 2002, ebolavirus RNA was detected in three forest-dwelling species of fruit bat in Gabon during an investigation which followed outbreaks of EHF [11] and in 2005 nucleic acid of Marburg virus was detected in *R. aegyptiacus* bats in the same country in the absence of a corresponding outbreak of disease [12]. On both occasions it again proved impossible to isolate live virus.

In July 2007, a small outbreak of MHF occurred in workers mining lead and gold in Kitaka Cave near Ibanda village in western Uganda. Large numbers of *R. aegyptiacus* and insectivorous *Hipposideros species* bats were present in this mine. Ecological investigations were conducted in August 2007 and May 2008, and the findings are presented here.

## Results/Discussion

### Identification of MHF in Kitaka miners

Kitaka Cave was first mined in the 1930s and eventually became a large producer of lead ore in Uganda, but was closed in 1979. It was reopened in January 2007, and in July a miner working in the cave fell ill and died with disease confirmed at Centers for Disease Control and Prevention, USA (CDC) to be MHF (patient A, Table 1). The Ugandan Ministry of Health closed the mine shortly thereafter. Following the month-long ecological investigation in August 2007, a second miner (patient B, Table 1) was confirmed to have MHF. The timing of his onset of symptoms in September, plus a lack of epidemiologic linkage to the first case, suggested that he re-entered the mine surreptitiously shortly after the departure of the investigating team. Thus, it appears that the ecological study was conducted at a time when Marburg virus activity was continuing. Marburg virus was isolated from each of the two miners, and full-length genome sequences were determined (01Uga2007 and 02Uga2007 respectively). Retrospective analysis of Patient A's contacts found two additional Kitaka miners positive for Marburg virus-specific IgG (data not shown). Both of these miners reported symptoms consistent with MHF in the month prior to Patient A falling ill.

**Table 1:** Summary of Marburg virus diagnostic test results for samples sent to CDC from patients A and B.

Patient	Sample ID No.	Days post onset	Ag	IgG	Q-RT-PCR (Ct )	NP PCR	VP35 PCR	L PCR	Isolation
**A**	200702854	NA	Pos	Neg	Pos (22)	Pos	Pos	Pos	Pos
**B**	200703648	7	Neg	Neg	Pos (32)	Pos	Pos	Neg	Pos
**B**	200703658	10	Neg	Neg	Pos (34)	NA	NA	NA	NA
**B**	200706136	20	Neg	Pos	Neg	NA	NA	NA	NA

### Detection of Marburg virus by Q-RT-PCR, virus isolation, IgG ELISA, and immunohistochemistry in bats found in Kitaka Cave

Marburg virus nucleic acid was detected by Q-RT-PCR in a total of 32 bats, and for the first time, live virus was isolated from five of the bats (Tables 2 and 3). There was a direct correlation between RNA levels (viral load) determined by Q-RT-PCR and the ability to isolate virus; 4/5 bats which yielded isolates had the highest RNA levels (lowest Ct values) (Table 3). Although rigorous quantitative analysis was not performed, the highest viral load measured (a Ct value of 24 recorded in bat 371), if compared to a liquid sample, corresponded to an approximate infectious titer of 1×10^5^ pfu/ml. This suggests that some infected individuals contain high levels of virus and may be shedding, perhaps infecting other animals, including humans. The fact that four isolates were obtained from *R. aegyptiacus* bats caught in 2007 and the fifth isolate came from a bat of the same species caught nine months later in 2008 implies that *R. aegyptiacus* colonies can harbor Marburg virus for extended periods of time. Previous studies [10],[11],[12] indicated that a modest prevalence of low-titered virus could be expected in liver and spleen samples. Possible reasons for the success in isolating live virus in the present study include the fact that an effort was made to sample relatively large numbers of bats and to flash freeze and preserve samples in liquid nitrogen directly after dissection. Moreover, the limited size of the outbreak in humans allowed the investigators to concentrate on implementing the initial ecological study shortly after the outbreak started, while virus activity in the bat colony was probably still high.

**Table 2:** Summary of species, gender and age of all bats captured and tested from the August 2007 and April–May 2008 collections.

Collection	Species	Total	No. PCR positive	% of total
August '07	*R. aegyptiacus*	411	22	5.6
	Male	184	8	5.7
	Female	226	14	5.5
	Female (pregnant)	182	4	2.1
	Adult	333	14	4.2
	Juvenile	78	8	10.3
	*Hipposideros* spp.	407	1	0.2
	Male	198	0	ND
	Female	209	1	ND
April–May '08	*R. aegyptiacus*	200	9	4.5
	Male	84	6	7.1
	Female	116	3	2.5
	Adult	140	8	5.7
	Juvenile	60	1	1.6
	*Hipposideros* spp.	202	0	ND
	Male	87	0	ND
	Female	115	0	ND

Listed by species is the total number of bats for each gender or age classification, with the percentage of Marburg virus positive bats (by Q-RT-PCR) within each classification listed in the column to the right.

**Table 3:** Summary of all Marburg virus positive bats in each collection period.

Collection	Bat No.	Species	Sex	Status	Ct	RT-PCR NP-VP35	Virus isolation	Sample ID/ Virus isolate No.
August '07	**44**	*R. aegyptiacus*	F	Adult	35.0	**Yes**	**Yes**	**200704525/811274**
	**77**	*R. aegyptiacus*	F	Adult	38.2			
	**97**	*R. aegyptiacus*	M	Adult	38.7			
	**188**	*R. aegyptiacus*	F	Adult	28.6	**Yes**	**Yes**	**200704669/811275**
	**208**	*R. aegyptiacus*	F	Adult (Preg)	39.4			
	**209**	*R. aegyptiacus*	F	Adult	38.8			
	**238**	*R. aegyptiacus*	M	Adult	39.4			
	**273**	*R. aegyptiacus*	M	Adult	35.0			
	**276**	*R. aegyptiacus*	M	Adult	39.6	**Yes**		
	**278**	*R. aegyptiacus*	F	Adult (Preg)	39.3			
	**288**	*R. aegyptiacus*	F	Juvenile	35.0	**Yes**		
	**291**	*R. aegyptiacus*	F	Juvenile	35.9	**Yes**		
	**311**	*R. aegyptiacus*	F	Adult w/pup (neg)	38.7			
	**323**	*R. aegyptiacus*	F	Adult (Preg)	36.8			
	**328**	*R. aegyptiacus*	M	Juvenile	30.7	**Yes**		
	**331**	*R. aegyptiacus*	M	Juvenile	29.1	**Yes**	**Yes**	**200703992/811276**
	**371**	*R. aegyptiacus*	F	Juvenile	24.0	**Yes**	**Yes**	**200704852/811277**
	**374**	*R. aegyptiacus*	F	Juvenile	34.4			
	**427**	*Hipposideros spp*	F	Adult	32.0	**Yes**		
	**721**	*R. aegyptiacus*	M	Adult	37.1			
	**756**	*R. aegyptiacus*	F	Adult (Preg)	38.6			
	**772**	*R. aegyptiacus*	F	Juvenile	37.1	**Yes**		
	**782**	*R. aegyptiacus*	M	Juvenile	36.9	**Yes**		
April '08	**839**	*R. aegyptiacus*	F	Adult	39.2			
	**883**	*R. aegyptiacus*	M	Juvenile	34.8	**Yes**		
	**901**	*R. aegyptiacus*	F	Adult	38.8			
	**924**	*R. aegyptiacus*	M	Adult	39.4			
	**931**	*R. aegyptiacus*	F	Adult	39.5			
	**946**	*R. aegyptiacus*	M	Adult	36.9			
	**982**	*R. aegyptiacus*	M	Adult	31.8	**Yes**	**Yes**	**200805444/811391**
	**989**	*R. aegyptiacus*	M	Adult	38.5			
	**1013**	*R. aegyptiacus*	M	Adult	35.0	**Yes**		

Listed for each bat is the species, sex, status and specific Q-RT-PCR, conventional RT-PCR (NP and VP35), and virus isolation test result. Shown in the far right column are the unique identification numbers for the tissues from which virus was isolated. Note that Marburg virus was isolated from liver/spleen tissues that tended to have the highest viral loads (lower Ct values) as measured by Q-RT-PCR.

By equating RNA-positivity with virus infection, it is possible to derive preliminary conclusions on the dynamics of Marburg virus activity in bat populations. Although there was a similar frequency of RNA-positivity in bats collected in August 2007 and May 2008, the fact that a total of 31/611 (5.1%) *R. aegyptiacus* bats in both collections tested positive in comparison to only 1/609 (0.2%) *Hipposideros spp. bat* (Table 2), suggests that infection in the latter species represented spillover from circulation of virus in *R. aegyptiacus* bats. In contrast, approximately equal proportions (3.0–3.6%) of *R. aegyptiacus* and two species of insectivorous bats were found positive for Marburg virus RNA in Goroumbwa Mine, DRC, in 1999 [10],[11],[12], but meaningful comparisons are precluded by differences in sample size and the inadequacy of population estimates.

Serologic testing found 13/546 (2.4%) *R. aegyptiacus* bats (data not shown), all adults, clearly positive for Marburg virus-specific IgG antibody (titer≥400, sum OD>0.95), two of which (#s 273 and 278) were also weakly positive by Q-RT-PCR. The testing found 455/546 (83.3%) bats to be clearly negative, while another 78 *R. aegyptiacus* bats had indeterminate antibody levels (titer = 100, sum OD≥0.33≤0.95). None of the *Hipposideros spp.* bats had detectable IgG to Marburg virus. It is unclear why only a low percentage of the *R. aegyptiacus* bat population is found positive for Marburg virus reactive IgG antibody. Perhaps a greater proportion of the population was previously infected, but antibody levels are below the conservative IgG assay cut-off used here. This would be consistent with low Marburg virus reactive antibody levels reported in previous bat studies [10],[12]. The finding that only 2/13 IgG positive bats had detectable virus nucleic acid would suggest the majority of virus is being cleared prior to Marburg virus reactive antibody becoming detectable.

All bats caught in 2007 and 2008 appeared healthy enough to leave their roosts to forage for food, the ratio of male to female *R. aegyptiacus* was similar in the two collections, and there appeared to be no gender bias in the evidence for Marburg virus infection (Table 2). However, the proportions of *R. aegyptiacus* juveniles and pregnant females present in the 2007 and 2008 collections differed markedly, and this appears to be consistent with the fact that the species is known to give birth in March and September in Uganda [13],[14]. After a gestation period of 105–7 days, females usually give birth to a single pup which is carried attached to a nipple on the female for 6 weeks, then left at the roosting site and fed with regurgitated food for 9–10 weeks, before flying and fending for itself [15]. Thus, in August 2007, 182/226 (80.5%) *R. aegyptiacus* females were found to be pregnant ahead of giving birth in September, and juveniles, mostly weaned, represented 78/411 (19%) of the collection. The prevalence of Marburg virus RNA detected in the juveniles, 8/78 (10.3%), was significantly higher than in adult *R. aegyptiacus* bats, 14/333 (4.2%) (p<.05, Fisher's exact test; Table 2). Only 4/182 (2.1%) of the pregnant females were RNA-positive, and their placentas all tested negative. Additionally, a single RNA-positive mother nursing an RNA-negative newborn pup was identified. In May 2008, no *R. aegyptiacus* females were found to be pregnant, although microscopic examination of uterine tissues were not performed, and juveniles, presumably born mostly in March, represented 60/200 (30%) of the collection, but only 1/60 (1.6%) of the juveniles were RNA-positive (Table 2).

It can be concluded that there was no evidence of vertical transmission of infection in *R. aegyptiacus*, but that juveniles are exposed to virus at a stage of their development possibly determined by factors such as waning maternal immunity or seasonal occurrence of infection in external hosts such as arthropods. Limited tests on arthropod parasites of bats in the present study were negative for evidence of Marburg virus infection (data not shown), and the same was true for larger numbers of parasitic and cave-associated arthropods tested in the investigations in the DRC in 1999 [10]. It seems more likely that there is horizontal transmission of infection among susceptible bats, as was proposed for Hendra virus [16] and Nipah virus [17]. However, no Marburg virus RNA was detected in oral swabs taken from bats, including those with virus RNA-positive liver and spleen samples (data not shown), suggesting that transmission via masticated fruit spats as suggested for Nipah virus, is an unlikely route for Marburg virus. Transmission via bat urine or feces would be another possible mechanism. It is notable that ebolavirus was found to be shed in the feces of experimentally infected fruit bats for up to 3 weeks [8], but limited immunohistochemical analyses of formalin-fixed kidneys of our RT-PCR positive bats have thus far been negative, tentatively suggesting that transmission via urine may be less likely than through feces. However, it would be premature to rule out transmission though urine, feces or saliva given the limited number of bats tested to date, and the lesser sensitivity of immunohistochemical methods relative to RT-PCR. The determination of virus transmission mechanisms will be best addressed in the future through experimental infection of *R. aegyptiacus* bats.

For ebolavirus, it has been suggested that outbreaks in nonhuman primates follow seasonal patterns which may reflect changes in diet or reproductive status of reservoir hosts, and that infection of the primates could be initiated through consumption of fruit contaminated with blood and placentas during parturition of infected bats [18],[19]. Our data indicating the lack of evidence for vertical transmission of Marburg virus would suggest blood and placentas generated during parturition are unlikely to be source of virus infecting primates, at least for Marburg virus.

**Figure 1:**
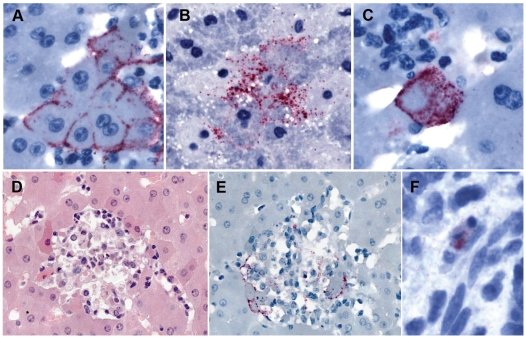
Immunohistochemical localization of Marburg virus antigens in *Roussetus aegypticus* tissues. In the liver, viral antigens were distributed in and around hepatocytes in a dense (A) or loose (B) perimembranous pattern. Rarely, entire hepatocytes were involved (C). These infected foci were characteristically sparse and were often associated with small collections of mononuclear inflammatory cells and hepatocyte necrosis (D and E), although infected cells could also be identified without conspicuous inflammatory infiltrates. Only rare viral antigens were seen in a few mononuclear cells of the spleen of 1 bat (F). Immunoalkaline phosphatase with napthol fast-red and hematoxylin counterstain (A–C, E, F), and hematoxylin and eosin (D); original magnifications ×100 (A, B, D, E) and ×258 (C, F).

Histopathological examination of liver and spleen samples of 30 *R. aegyptiacus* bats and one *Hipposideros spp.* bat which produced positive PCR results, and 49 bats which were uniformly negative in Q-RT-PCR plus NP and VP35 RT-PCR assays, revealed no lesions which could specifically or consistently be ascribed to Marburg virus infection. Viral antigens were detected by IHC in the livers of two bats which yielded Marburg virus isolates in culture (bats 331 and 371, Table 3) and were distributed predominantly in a perimembranous pattern around small, relatively isolated foci of hepatocytes. These foci were often associated with small accumulations of mononuclear inflammatory cells and highly localized hepatocyte necrosis (Figures 1A–E). Rare Marburg virus antigens were observed in the spleen of only one bat, number 371, and were localized to the cytoplasm of isolated mononuclear cells (Figure 1F). This represents the first time that filovirus antigens have been visualized in tissues of naturally infected bats. From the sparse and highly focal nature of the infected sites, it can be surmised that the methods used to sample and test bats, including the Q-RT-PCR, are likely to produce underestimates of the prevalence of active infection. The paucity of hepatic lesions and viral antigens detected by IHC in wild-caught *R. aegyptiacus* contrasts markedly with the abundant and extensively distributed Marburg virus antigens observed in the livers of infected humans and non-human primates [20],[21]. The histopathologic and immunohistochemical findings of Marburg virus infection in these naturally infected *R. aegyptiacus* are consistent with observations made for hemorrhagic fever viruses of the families *Arenaviridae*, *Bunyaviridae*, and *Paramyxoviridae* in their small-mammal reservoir hosts [22],[23],[24], and lend additional support to the contention that *R. aegyptiacus* is a reservoir for Marburg virus.

### Estimation of *R. aegyptiacus* colony size

During the 2008 field trip a total of seven of 1,329 marked bats at the Kitaka mine were recaptured at a rate of about 1% of total nightly catches (data not shown), and from these data it was calculated that approximately 112,000 *R. aegyptiacus* bats roosted in Kitaka mine. By extrapolation from the approximately 5% viral RNA-positive levels detected by Q-RT-PCR in the bats tested in 2007 and 2008, it follows that there could be >5,000 infected bats within the colony at any one time, suggesting that there is a high risk of infection for humans who spend extended periods in close proximity to the bats. In fact, in December 2007 and again in July 2008, an American and Dutch tourist acquired non-fatal and fatal Marburg virus infections respectively after encountering *R. aegyptiacus* bats in Python Cave in the Queen Elizabeth National Park, <30 miles from Kitaka mine [25],[26].

### Phylogenetic analysis of Marburg virus sequences from bats and miners

**Figure 2:**
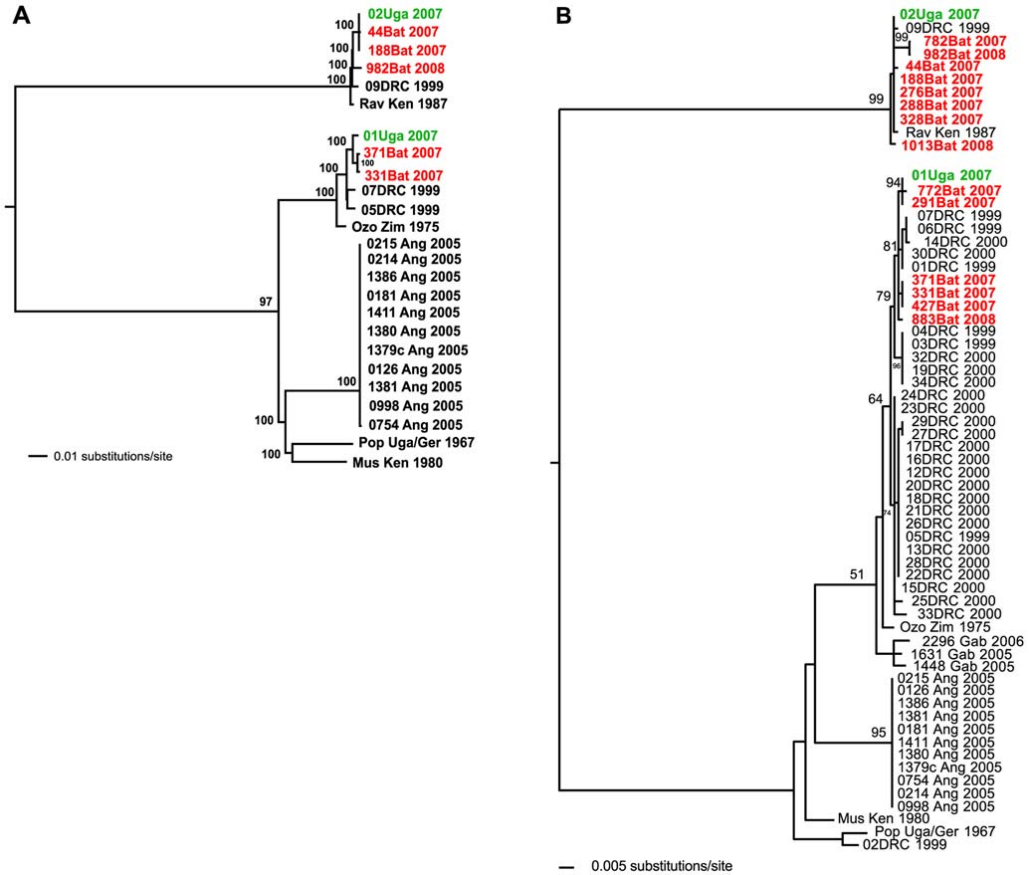
Phylogenetic analysis of full-length or partial genomes of Marburg viruses isolated from humans or bats (see Table S1 for Genbank accession numbers). Trees shown are maximum-likelihood analyses with Bayesian posterior probabilities >50 listed at the appropriate nodes. The ebolavirus outgroup used during the Bayesian phylogenetic analyses are denoted by the small twig at the root of the tree. Marburg virus sequences from 2007 human cases in Uganda are in green, while those from bats are listed in red. (A) Analysis of full-length genomes of five Marburg virus bat isolates, 18 historical isolates, and the isolates from patients A and B (01Uga07 and 02Uga07 respectively). (B) Phylogenetic analysis of concatenated NP and VP35 sequence fragments obtained from each bat specimen compared to corresponding regions from 48 historical isolates and those from 01Uga07 and 02Uga07.

The results of Bayesian analysis of the nucleotide differences among full-length virus genome sequences of the isolates from the two miners (01Uga2007 and 02Uga2007), plus the five isolates from bats (44, 188, 331, 371 and 982, Table 3), and 18 representative historical Marburg virus isolates, is shown in Figure 2A. Isolate 01Uga2007 falls into the prototypic clade containing the majority of known Marburg virus sequences. The second human isolate, 02Uga2007, which differs by 21% (nucleotide level) from 01Uga2007, is closely related to members of the highly distinct Ravn lineage, first isolated in 1987 from a patient (RavKen1987) who ostensibly acquired infection in Kitum Cave, Kenya [5]. Thus, it is clear that the Kitaka mine outbreak represented two independent introductions of infection from the natural reservoir hosts into the human population. Two of the bat isolates group with the majority of historical Marburg virus sequences and are most closely related (99.3% identical) to the sequence from miner A (01Uga2007), while the other 3 bat isolates reside within the Ravn lineage (RavKen1987) and are closely related (99.2–99.9% identical) to the sequence from miner B (02Uga2007).

In order to extend the phylogenetic analysis to virus RNA-positive bats from which no isolates were obtained, concatenated partial NP and VP35 gene sequences determined for 14 bats during the present study, plus 2 equivalent sequences derived from the human isolates, and 48 sequences derived from data for historical Marburg virus isolates (Genbank accession numbers in Table S1), were subjected to Bayesian analysis (Figure 2B). No sequences could be determined for a further 17 bats which were positive for viral RNA by Q-RT-PCR, possibly because the viral loads were too low for conventional NP and VP35 RT-PCR to detect. Nevertheless, it was clear that diverse Marburg virus lineages were circulating in the Kitaka mine bats, and that some were identical or near-identical to the human isolates across the genome fragments examined. Sequences from bats 291 and 772 were either identical or within one nucleotide, respectively, of isolate 01Uga2007 (miner A), while sequences from bats 44, 188, 276, 288 and 328 closely matched 02Uga2007 (miner B). The identification of virus lineages circulating in bats within Kitaka mine was probably incomplete, but even these limited genetic data suggest recent common ancestry for closely matching genomes found in bats and humans and strongly implicate *R. aegyptiacus* as the primary source of human infection. The structure of the outbreak was strikingly similar to that seen in 1999 in Durba, DRC, as that outbreak also involved multiple introductions of virus from the natural reservoir, putatively bats, into the human population, plus the co-circulation of highly divergent Marburg viruses in a single geographic location [9],[10].

### Concluding remarks

The generation and perpetuation of such diverse genetic lineages of virus, with ≥21% nucleotide differences, imply the need for a long association of the virus with its reservoir host, plus the need for a large host population with constant recruitment of naïve individuals. The estimated population of 112,000 *R. aegyptiacus* bats in Kitaka mine could probably produce up to 100,000 offspring with two breeding seasons a year. Moreover the species is widely distributed in Africa, with many large colonies in proximity in East Africa alone, including the Kitum Cave complex on Mount Elgon, and numerous caves in western Uganda. It has been observed in South Africa that large proportions of the bats within *R. aegyptiacus* colonies migrate ≥300 miles to other colonies on a seasonal basis [27]. Hence the potential pool of vertebrate hosts for Marburg virus may extend to tens of millions of bats across a large geographic range.

Although diverse Marburg virus lineages were found to co-circulate at single geographic locations in Kitaka mine in Uganda and Goroumbwa Mine in the DRC, it is noteworthy that very closely related lineages have also been found at widely separated geographic locations, in some instances over 2000 km apart. For example, Marburg virus sequences found in bats in Gabon are closely related to isolates from Zimbabwe, Uganda and DRC. Isolates of the Ravn lineage have been found in Kenya, DRC and Uganda. In fact, an isolation-by-distance analysis of the data presented here (Mantel test) found no correlation between genetic and geographic distances (p>0.3). The geospatial separation of the closely related Marburg virus lineages is most consistent with mobility of their natural host, a dynamic easily accomplished by the enormous meta-population of *R. aegyptiacus* present in Africa.

Longitudinal studies of naturally infected *R. aegyptiacus* colonies would provide valuable insights into the dynamics of immune status, as well as the shedding, transmission and persistence of Marburg virus in bat populations, and help to determine if the proportions of infected individuals relative to age are periodic or stochastic. The studies should be supplemented by experimental infections to observe the dynamics of infection within individual bats. Given the detection of infectious ebolavirus in privileged sites, such as testes, up to three months after onset of symptoms in human infections [28], careful examination of multiple tissues from infected bats is also warranted.

## Materials and Methods

### Human samples

Blood samples collected during acute illness and submitted as diagnostic samples to the Centers for Disease Control and Prevention (CDC), Atlanta, USA, were tested for Marburg virus antigen and IgG antibody by enzyme-linked immunoassay as described previously [29],[30]. The samples were also tested for presence of Marburg virus nucleic acid by reverse transcriptase-polymerase chain reaction (RT-PCR), and cultured for isolation of virus as described below.

### Bat samples

According to an institutionally reviewed IACUC protocol, bats were captured with mist nets or harp traps at the opening of the mine, euthanized with Isoflurane, and samples of liver, spleen and placenta (where applicable) collected by dissection, using safety precautions described previously [31]. Liver and spleen were selected for sampling based upon previous studies [10],[11],[12] and because these organs are affected in filovirus infections of primates. Aliquots of tissue were preserved in chaotrope (Cellular Lysis Buffer, Applied Biosystems) for analysis by RT-PCR, while replicate samples were frozen in liquid nitrogen for culture of virus, and fixed in formalin for histopathological examination. Blood was also taken from each bat for RT-PCR and antibody analyses as described below. Bats were identified morphometrically [32], their measurements and breeding status recorded, and the carcasses preserved in 10% formalin for at least 1 week and later changed to 70% ethanol for long-term storage. To minimize the potential for cross-contamination between bat samples, all dissection instruments were used only once during each nightly necropsy session, and in between sessions, all instruments were soaked in 3% Lysol for ≥15 minutes followed by disinfection in 10% bleach for ≥15 minutes.

To maximize the chances of isolating virus, large numbers of each of the two species of bat found in the mine, the fruit bat *R. aegyptiacus* and the insectivorous *Hipposideros* spp. bats, were sampled during the first field trip in August 2007. Smaller numbers were sampled during the second field trip which was undertaken in May 2008, during the putative breeding season of *R. aegyptiacus* bats in Uganda, mainly to seek evidence of continued circulation of virus and possible vertical transmission of infection. Opportunity was taken to collect oral swabs from the bats sampled in May to determine the likelihood of virus transmission through saliva or respiratory aerosols. A mark and recapture study was also conducted in May to estimate the size of the *R. aegyptiacus* population, and to possibly allow for later determination of foraging and migration distances of the bats. A total of 1,329 *R. aegyptiacus* bats were tagged with coded aluminum necklaces or leg bands over a period of two weeks, and recaptures which were recorded once the number of marked bats reached 1,000, were used in the Jolly-Seber model for estimating the abundance of an open population [33],[34].

### Collection of additional fauna within the mine

Limited numbers of arthropod parasites of bats were collected and frozen, including 25 wingless flies (Family *Nycteribiidae*) found in the pelage of bats during dissection, and 100 adult and nymphal argasid ticks (*Carios faini*) taken from crevices in the rocks near bat roosting sites. Apart from dermestid beetles, spiders, crickets, moth flies and cockroaches, the only other fauna seen in the cave consisted of a target rat (*Stochomys longicaudatus*) and forest cobras (*Naja melanoleuca*).

### RT-PCR analysis

Total RNA was extracted in one of two ways. 50 µ liquid samples (blood and eluates of oral swabs) were extracted using non-cellular lysis buffer (Applied Biosystems) [35] while RNA from tissue (100 mg) were extracted with cellular lysis buffer (Applied Biosystems) [12]. RT-PCR based assays for the NP, VP35 and VP40 genes, were performed as described previously [9],[12],[36], except that the VP40 quantitative RT-PCR assay (Q-RT-PCR) assay was modified to include two reporter-labeled probes 5′Fam-ATCCTAAACAGGC“T”TGTCTTCTCTGGGACTT-3′ and 5′Fam-ATCCTGAATAAGC“T”CGTCTTCTCTGGGACTT-3′ in addition to the forward primer 5′-GGACCACTGCTGGCCATATC-3′and reverse primer 5′-GAGAACATITCGGCAGGAAG-3′. The quencher BHQ1 was placed internally in the probes at the “T” sites.

All human and bat samples were screened by Q-RT-PCR, designed to detect RNA of all known lineages of Marburg virus, and bat samples found positive (Ct<40) were re-analyzed by extracting RNA from frozen tissue using RNAeasy mini-kits (Qiagen) after overnight incubation at 4°C in lysis buffer. The extracts were subjected to the Q-RT-PCR and conventional RT-PCR based on the NP and VP35 genes. Tissues from 39 bats found negative in the initial Q-RT-PCR were also re-extracted and subjected to Q-RT-PCR and NP and VP35 gene RT-PCR. Nycteribid flies and argasid ticks were individually ground in cellular lysis buffer and extracted RNA tested by Q-RT-PCR.

### Virus isolation

For human samples, 100 µl of blood was inoculated onto Vero E6 monolayers in 25 cm^2^ flasks and incubated for 14 days at 37°C/5% CO_2_ in MEM/2% fetal calf serum with a media change after day 7. Cultures were monitored daily for CPE with cell scrapes at days 7 and 14 tested by IFA. For bat samples, 10% suspensions of freshly thawed ∼250 mg frozen tissue sections were homogenized on ice in viral transport medium (HBSS/5% fetal calf serum) with a plastic pestle and ∼250 mg sterile alundum (Fisher cat# A634-3) in 15 ml conical tubes. The homogenate was clarified by low speed centrifugation and 100 µl of supernatant fluid was inoculated onto Vero E6 cell cultures in 25 cm^2^ flasks at 37°C/5% CO_2_ for 1 hr with gentle rocking followed by media replacement with MEM/2% fetal calf serum. Inoculated flasks were monitored daily for 14 days (with media change after day 7) for the appearance of CPE and by IFA of cell scrapes on days 7 and 14. Cultures positive by IFA for Marburg virus were additionally analyzed by RT-PCR (see below).

### Nucleotide sequencing of PCR products and virus isolates

Sequencing of Marburg virus whole genomes and partial gene sequences (NP and VP35) were performed as previously described [12],[36].

### IgG detection in bats

Blood samples from bats were tested by enzyme-linked immunoassay for the presence of IgG antibody reactive with Marburg virus as described previously [29],[30] but with the following modifications: 1) 96-well plates were coated with Marburg virus infected cell lysate (diluted 1∶1000 final concentration) generated from Marburg virus isolates # 188 (Ravn lineage) and #371 (main lineage), 2) sera were initially diluted 1∶100 in 5% nonfat milk rehydrated in PBS-T containing normal Vero E6 cell slurry diluted 1∶25 and then further diluted 4-fold through 1∶6400 in PBS-T/5% nonfat milk, and 3) bound bat-specific IgG was detected using HRP-conjugated goat anti-bat IgG (Bethyl-L cat# A140-118P) diluted 1∶2000. The mean and SD of the adjusted sum ODs from the entire collection (both species) were used to plot a frequency distribution and calculate a value greater than the mean+3 SD. Sera with repeatable adjusted sum ODs greater than this cutoff value (0.95) and whose titers were ≥1∶400 were considered positive.

### Phylogenetic analyses

Genbank accession numbers are described in Table S1. Phylogenetic analyses were performed separately on two sets of data: one comprising 25 whole genome sequences including those of 18 representative historical Marburg isolates, plus the 2 isolates obtained from miners and 5 isolates obtained from bats during the present investigations, and the second data set was comprised of 64 concatenated partial NP and VP35 gene sequences including 48 derived from historical Marburg isolates plus 2 derived from the isolates obtained from miners and 14 determined for PCR products obtained from bats during the present study. A representative sample of Ebola Zaire (Genbank accession NC 002549) was used as an outgroup.

Modeltest 3.730 [37] was used to examine 56 models of nucleotide substitution to determine the model most appropriate for the data. For whole genome analysis, the General Time Reversible model incorporating invariant sites and a gamma distribution (GTR+I+G) was selected using the Akaike Information Criterion (AIC). Nucleotide frequencies were A = 0.326, C = 0.195, G = 0.185, T = 0.294, the proportion of invariant sites = 0.451, and the gamma shape parameter = 7.244. The Kimura 3-parameter model with unequal base frequencies and a proportion of invariant sites (K81uf+I) was selected for the concatenated NP-VP35 dataset. Nucleotide frequencies were A = 0.310, C = 0.233, G = 0.202, T = 0.255, and the proportion of invariant sites = 0.659. Maximum likelihood analyses were subsequently performed in PAUP*4.0b10 [38] using the GTR+I+G or K81uf+I model parameters.

In addition, Bayesian phylogenetic analyses were conducted for each of the datasets in MrBayes 3.2 [39] using the GTR+I+G model of nucleotide substitution. For each dataset, two simultaneous analyses, each with four Markov chains, were run for 10,000,000–40,000,000 generations, sampling every 100 generations. Prior to termination of the run, the AWTY program was used to assess convergence to ensure that the length of the analysis was sufficient [40]. Trees generated before the stabilization of the likelihood scores were discarded as burn-in, and the remaining trees were used to construct a consensus tree. Nodal support was assessed by posterior probability values (≥95 = statistical support).

### Histopathological examination of bat tissues

To determine if marburg virus infection caused lesions in infected bats, sections were cut from paraffin-embedded blocks prepared from formalin-fixed liver and spleen samples from 32 bats found positive by Q-RT-PCR, and examined in parallel with the tissues of 39 bats found negative in both the Q-RT-PCR and conventional RT-PCR. Hematoxylin and eosin (H&E) stained sections of the tissues were examined for lesions, and sections stained by an immunoalkaline phosphatase technique [41] with a polyclonal rabbit anti-Marburg virus antiserum diluted to 1/1000. Samples were evaluated without prior knowledge of the PCR and virus culture results.

## Supporting Information

Table S1 Genbank Accession numbers used for phylogenetic analysis
(0.08 MB DOC)
