# Supplementary material for: Isolation of Genetically Diverse Marburg Viruses from Egyptian Fruit Bats
Source: PLoS Pathog. 2009 Jul 31;5(7):e1000536. doi: 10.1371/journal.ppat.1000536 (PMC2713404; doi:10.1371/journal.ppat.1000536)
Supplement: Table S1 — Genbank Accession numbers used for phylogenetic analysis (0.08 MB DOC) [file ppat.1000536.s001.doc]

**Supplementary online material**

**Table S1**

| **Sequence** | **NP-fragment** | **VP35-fragment** | **Whole genome** |
| --- | --- | --- | --- |
| **01Uga07** |  |  | **FJ750957** |
| **02Uga07** |  |  | **FJ750953** |
| **44Bat 2007** |  |  | **FJ750954** |
| **188Bat 2007** |  |  | **FJ750955** |
| **982Bat 2008** |  |  | **FJ750956** |
| **331Bat 2007** |  |  | **FJ750959** |
| **371Bat 2007** |  |  | **FJ750958** |
| **782Bat 2007** | **FJ743669** |  |  |
| **276Bat 2007** | **FJ743670** | **FJ743678** |  |
| **288Bat 2007** | **FJ743672** | **FJ743680** |  |
| **328Bat 2007** | **FJ743674** | **FJ743682** |  |
| **1013Bat 2008** | **FJ743676** | **FJ743684** |  |
| **772Bat 2007** | **FJ743671** | **FJ743679** |  |
| **291Bat 2007** | **FJ743673** | **FJ743681** |  |
| **427Bat 2007** | **FJ743675** | **FJ743683** |  |
| **883Bat 2008** | **FJ743677** | **FJ743685** |  |
| **01DRC 1999** | **FJ743650** | **DQ466112** |  |
| **02DRC 1999** | **FJ743668** | **DQ466116** |  |
| **03DRC 1999** | **FJ743643** | **DQ466110** |  |
| **04DRC 1999** | **FJ743642** | **DQ466111** |  |
| **05DRC 1999** |  |  | **DQ447651** |
| **06DRC 1999** | **FJ743647** | **DQ466114** |  |
| **07DRC 1999** |  |  | **DQ447650** |
| **09DRC 1999** |  |  | **DQ447652** |
| **12DRC 2000** | **FJ743657** | **DQ466120** |  |
| **13DRC 2000** | **FJ743662** | **DQ466122** |  |
| **14DRC 2000** | **FJ743648** | **DQ466125** |  |
| **15DRC 2000** | **FJ743664** | **DQ466121** |  |
| **16DRC 2000** | **FJ743656** | **DQ466123** |  |
| **17DRC 2000** | **FJ743655** | **DQ466124** |  |
| **18DRC 2000** | **FJ743659** | **DQ466126** |  |
| **19DRC 2000** | **FJ743645** | **DQ466127** |  |
| **20DRC 2000** | **FJ743658** | **DQ466128** |  |
| **21DRC 2000** | **FJ743660** | **DQ466129** |  |
| **22DRC 2000** | **FJ743665** | **DQ466130** |  |
| **23DRC 2000** | **FJ743652** | **DQ466132** |  |
| **24DRC 2000** | **FJ743651** | **DQ466131** |  |
| **25DRC 2000** | **FJ743666** | **DQ466143** |  |
| **26DRC 2000** | **FJ743661** | **DQ466133** |  |
| **27DRC 2000** | **FJ743654** | **DQ466134** |  |
| **28DRC 2000** | **FJ743663** | **DQ466135** |  |
| **29DRC 2000** | **FJ743653** | **DQ466136** |  |
| **30DRC 2000** | **FJ743649** | **DQ466137** |  |
| **32DRC 2000** | **FJ743644** | **DQ466139** |  |
| **33DRC 2000** | **FJ743667** | **DQ466141** |  |
| **34DRC 2000** | **FJ743646** | **DQ466142** |  |
| **0215 Ang 2005** |  |  | **DQ447658** |
| **0126 Ang 2005** |  |  | **DQ447656** |
| **1386 Ang 2005** |  |  | **DQ447655** |
| **1381 Ang 2005** |  |  | **DQ447654** |
| **0181 Ang 2005** |  |  | **DQ447653** |
| **1411 Ang 2005** |  |  | **DQ447653** |
| **1380 Ang 2005** |  |  | **DQ447653** |
| **1379c Ang 2005** |  |  | **DQ447653** |
| **0754 Ang 2005** |  |  | **DQ447659** |
| **0214 Ang 2005** |  |  | **DQ447657** |
| **0998 Ang 2005** |  |  | **DQ447660** |
| **2296 Gab 2006** | **EU068110** | **EU068113** |  |
| **1631 Gab 2005** | **EU068109** | **EU068112** |  |
| **1448 Gab 2005** | **EU068108** | **EU068111** |  |
| **Ozo Zim 1975** |  |  | **AY358025** |
| **Mus Ken 1980** |  |  | **DQ217792** |
| **Rav Ken 1987** |  |  | **DQ447649** |
| **Pop Uga/Ger 1967** |  |  | **Z29337** |

**Supplementary Table 1.** GenBank accession numbers of all Marburg virus sequences analyzed.
